# Immune Checkpoints in Cancers: From Signaling to the Clinic

**DOI:** 10.3390/cancers13184573

**Published:** 2021-09-12

**Authors:** Céline Pisibon, Amira Ouertani, Corine Bertolotto, Robert Ballotti, Yann Cheli

**Affiliations:** 1 Université Côte d’Azur, 06103 Nice, France; celine.pisibon@etu.univ-cotedazur.fr (C.P.); sarah.ouertani@etu.univ-cotedazur.fr (A.O.); Corine.bertolotto@univ-cotedazur.fr (C.B.); robert.ballotti@univ-cotedazur.fr (R.B.); 2 INSERM, Centre Méditerranéen de Médecine Moléculaire, Biology and Pathologies of Melanocytes, Team1, 06200 Nice, France

**Keywords:** immune checkpoint, immunotherapy, signaling, immune cells, melanoma, cancers

## Abstract

**Simple Summary:**

Immune checkpoint therapies are treatments used to fight cancers by reactivating a patient’s own immune system. Melanoma was the first cancer to benefit from these treatments. Despite a clear benefit for patients and the existence of long responders, most patients fail to respond or develop resistance to these treatments. In this review, we discuss immune checkpoint signaling in the different immune cells with their biological consequences and summarize new immune checkpoint therapies that are under investigation in clinical trials or in development to bypass resistances and to improve the outcome of these therapies.

**Abstract:**

The immune system is known to help fight cancers. Ten years ago, the first immune checkpoint inhibitor targeting CTLA4 was approved by the FDA to treat patients with metastatic melanoma. Since then, immune checkpoint therapies have revolutionized the field of oncology and the treatment of cancer patients. Numerous immune checkpoint inhibitors have been developed and tested, alone or in combination with other treatments, in melanoma and other cancers, with overall clear benefits to patient outcomes. However, many patients fail to respond or develop resistance to these treatments. It is therefore essential to decipher the mechanisms of action of immune checkpoints and to understand how immune cells are affected by signaling to be able to understand and overcome resistance. In this review, we discuss the signaling and effects of each immune checkpoint on different immune cells and their biological and clinical relevance. Restoring the functionality of T cells and their coordination with other immune cells is necessary to overcome resistance and help design new clinical immunotherapy strategies. In this respect, NK cells have recently been implicated in the resistance to anti-PD1 evoked by a protein secreted by melanoma, ITGBL1. The complexity of this network will have to be considered to improve the efficiency of future immunotherapies and may lead to the discovery of new immune checkpoints.

## 1. Introduction

**Figure 1:**
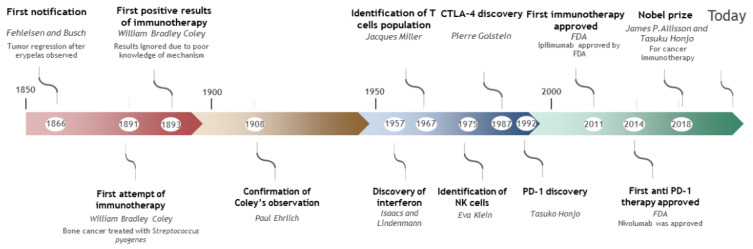
Development of immunotherapy.

After nearly a century of research, immunotherapy was established as an anticancer frontline treatment when the immune checkpoint inhibitor (ICI) ipilimumab (anti-CTLA-4) was approved in 2011 for melanoma treatment [1] (Figure 1). The immunotherapy concept started in the 19^th^ century with the observations of two German physicians who noticed significant tumor regression in a cancer patient after development of erysipelas, a skin infection caused by a bacterium, *Streptococcus pyogenes*. In 1891, William Bradley Coley, who is considered to be the father of immunotherapy, attempted to treat bone cancer by unleashing the immune system by infecting patients with bacteria responsible for erysipelas. He then extended this method to treat sarcoma, lymphoma and testicular carcinoma. Successful results were described in 1893 but were largely ignored by physicians due to poor knowledge about the underlying mechanisms. It was only in the mid-twentieth century that interest in the immune system to treat cancer reemerged with advances in immunity and the identification of interferon. In 1957, Burnet and Thomas proposed a cancer immuno-surveillance hypothesis based on the idea that the immune system can recognize and eliminate cancer cells [2]. However, due to a lack of strong experimental evidence, this concept failed to explain the prevalence of cancer. Thanks to the discovery of different immune cell populations such as T cells in 1967 and NK in 1975, the immuno-surveillance hypothesis was resurrected. Recent data showed that not only does immunosurveillance exist, but it is a part of a general process called immuno-editing [3]. By the end of the 1990s, this concept was largely supported by Robert Schreiber and Gavin Dunn, leaders in the immunosurveillance concept. Based on this concept, tumors are edited by the immune system, facilitating their escape by selecting for certain resistant variants [4], resulting in the development of the cancer due to insufficient immunosurveillance. Promoting the immune system became an interesting lead to explore. One of the first immunotherapy trials in metastatic cutaneous melanoma was approved by the FDA in 1998 for treatment of stage IV melanoma with IL-2 to stimulate white blood cell proliferation. However, only a 19% overall response rate was observed, with only a 4% complete response rate, limiting the use of this strategy [5]. Moreover, a high percentage of IL-2 treated patients developed vitiligo, an autoimmune disease resulting in skin depigmentation [6]. This observation supported the important role of the immune system in melanoma treatment.

Among the different populations of immune cells, cytotoxic T lymphocytes are often the final effectors against the tumor who detect and kill cancer cells. T cell development occurs in the thymus; immature cells proliferate and create a wide repertoire of T cell receptors (TCRs) through recombination of the TCR gene segments. A negative and positive selection process is then applied to eliminate T cells that react with themselves in order to prevent autoreactivity. T cells are considered naive before they encounter an antigen. At this point, a final clonal selection is applied in order to activate and expand specific T cells in secondary lymphoid tissues. This step is initiated through various ligand–receptor interactions between T cells and antigen-presenting cells (APCs); the first one being the antigen recognition by the TCR. The antigen is displayed with the major histocompatibility complex (MHC) on the APC surface. This process is regulated by a balance between co-stimulatory and inhibitory signals. In fact, T cells are controlled by immune checkpoint pathways to maintain self-tolerance and avoid excessive responses.

Another important population for immunosurveillance, but less specific than T cells, are natural killer (NK) cells [7,8,9]. This population is part of the innate immune system and unlike T cells, they do not require specific antigen binding to be activated [10]. Like T cells, a balance between co-stimulatory and inhibitory signals through specific surface receptors are needed to control NK activity.

The discovery and understanding of the immune checkpoint led to the development of immune checkpoint inhibitors (ICIs), whose action seems to potentiate the anti-tumor immune response and improve the survival of advanced-stage melanoma patients. Among the inhibitory checkpoints, cytotoxic T-lymphocyte-associated protein 4 (CTLA-4) and programmed cell death protein 1 (PD-1) are the most studied and are promising targets for cutaneous melanoma treatment. CTLA-4 was first discovered and identified by a French team from Marseille led by Pierre Golstein [11] while PD-1 was identified In Japan by Honjo T. and co-workers [12]. This novel treatment presented promising results as patients showed long-lasting remissions and ICIs have emerged as a frontline treatment in unresectable cutaneous metastatic melanoma. This new treatment offered new hope for some patients; however, a large percentage of individuals remain unresponsive to this treatment.

The Nobel Prize in Physiology or Medicine in 2018 was awarded to Professors James Allison and Tasuku Honjo for their research on CTLA-4 and PD-1, respectively. Our understanding of the immune checkpoint mechanism and signaling has come a long way; however, it is still incomplete and must be improved for advances to be made in this field.

In this review, we will provide an update on signaling pathways engaged by inhibitory immune checkpoints, their involvement with specific immune cell populations and their clinical relevance in the context of immunotherapies.

## 2. Immune Checkpoints Signaling

**Figure 2:**
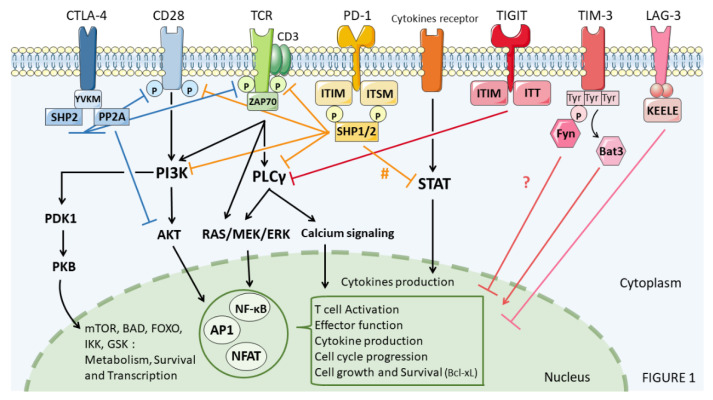
Immune checkpoint signaling in immune cells. # STAT inhibition after PD-1 recruitment remains to be carefully addressed.

In the following sections, we will focus on recent data regarding immune checkpoint signaling including CTLA-4, PD-1, LAG-3, TIM-3, TIGIT, VISTA and B7H3 (Figure 2).

### 2.1. CTLA-4, Cytotoxic T-Lymphocyte-Associated Protein 4

Cytotoxic T-lymphocyte-associated protein 4 (CTLA-4) is a transmembrane protein and a member of the CD28 family. This molecule is expressed on the surface of several activated immune cells including T cells, NK, iNKT and B cells. Its binding to CD80/CD86 induces an inhibitory signal.

The activation and proliferation of T cells requires the binding of TCR to MHC and calls for costimulatory signals. CD28 receptors on T-cells bind to B7 ligands (B7-1/CD80 and B7-2/CD86) on antigen-presenting cells (APCs) and provide the efficient activation signal for T-cells. CD28-CD80/86 binding increases tyrosine phosphorylation mediated by the SRC-family kinases. In addition, CD28-PI3K connection activates phosphoinositide dependent protein kinase 1 (PDK1) and protein kinase B (PKB⁄AKT). This activation phosphorylates different substrates [13] such as BAD, caspase-9, transcription factors CREB1 (cAMP responsive element binding protein 1) and forkhead, mTOR (mammalian target of rapamycin) and glycogen synthase kinases-3α and β (GSK3α and GSK3β, respectively) [13,14]. Therefore, CD28-B7 interaction induces T-cell proliferation and survival (by prosurvival factors Bcl-2 and Bcl-xL up-regulation) and increases cell metabolism and the production of growth cytokines such as interleukin-2 (IL-2), IFNɣ or IL-10 [15].

In case of chronic antigenic stimulation, such as chronic viral infection or cancer, a strong defect in CD8^+^ T cell responses is observed [16]. Indeed, CTLA-4 also binds CD80 and CD86 with a higher affinity, thus competing with CD28 and inducing anergy of T cells during the priming phase of the immune system [17].

A study has shown that an inhibitory signal may be related to the tyrosine-rich YVKM intracellular domain (Tyr-Val-Lys-Met, a YXXϕ motifs, where Y is tyrosine, X is any amino acid, and ϕ is an amino acid with a bulky hydrophobic side chain) on the CTLA-4 C-terminal [18]. The engagement of CTLA-4 with its ligands blocks the formation of TCR-ZAP70 micro clusters that prevent SLP-76 and LAT phosphorylation by ZAP70. This results in the inhibition of IL-2 and anti-apoptotic protein Bcl-xL expression, leading to decreased T cell activation, proliferation, and maturation. However, CTLA-4 does not interfere directly with ZAP-70 recruitment or phosphorylation [19,20].

CTLA-4 activation recruits SHP2 and PP2A phosphatases through the phosphorylated YVKM motif. Those phosphatases are responsible for the dephosphorylation of the tyrosine kinase and serine threonine kinase signaling pathway of TCR/CD28 [13] disrupting the activation of AKT, a key player in diverse cellular processes, cytokine synthesis and survival.

In CD4^+^ Treg, upregulation of CTLA-4 is associated with increased phosphorylation and hence activity of STAT3 in dendritic cells, resulting in a suppressed immune response [21]. Interestingly, the production of cytokines such as IFNγ, IL-17, and IL-22 was decreased while IL-4 was upregulated. A CD4-mediated increase in apoptosis was also observed [22,23]. In B cells, engagement of CTLA-4 with CD86 induced STAT3 activation through TIK2, increasing B cell lymphoma proliferation and tumor proliferation. Cytokine production was also dysregulated, showing an increase in IL-6 and IL-10 and a strong decrease in IFNγ expression. Finally, Treg CTLA-4 expression and binding to dendritic cells (DC) induce STAT3 activation by CD86 engagement, leading to decreased NF𝜅B signaling and the production of IFNγ. In NKT cells, upregulation of CTLA-4 leads to regulation of IFNγ through the mTOR pathway. Indeed, IFNγ production blockade by rapamycin treatment induces an immunosuppressive NKT phenotype [24].

Recent studies have highlighted the presence of CD28 and CTLA-4 on the surface of tumor-infiltrating NK cells in several mouse models of solid tumors. Their expression is thought to be upregulated by IL-2. As for T cells, CD28 is known to induce NK cell proliferation, cytotoxicity and cytokine secretion, producing large amounts of IFNγ which is in turn negatively regulated by CTLA-4 [25]. Moreover, additional data has established that anti-CTLA-4 therapy stimulates the activation and degranulation of NK cells in the tumor microenvironment (TME) in correlation with a depletion of intra-tumoral Tregs [26].

Yeast two-hybrid interaction analysis revealed that CTLA-4 associates with the μ2 subunit of the complex AP-2 mediating its internalization through the binding of the non-phosphorylated YVKM motif [27].

Therefore, non-phosphorylated CTLA-4 is internalized via its AP-2 association, whereas tyrosine phosphorylation triggers its stabilization at the cell surface, permitting CD80/86 engagement and negative signaling. This receptor is primarily localized in the intracellular vesicles and then released by exocytosis on the cell surface following the signal induced by TCR and CD28-B7 binding [15]. In addition, only a small fraction is exposed on the cell surface allowing CTLA-4 to present both cell-intrinsic and extrinsic functions [28]. Meanwhile Treg cells express CTLA-4 constitutively, and this molecule participates in the maintenance of tolerance applied by Treg cells [29,30].

Taken together, these observations implicate CTLA-4 during the priming phase, the first step of immune system activation against tumor and infection. CTLA-4 acts strongly on CD4 Treg to dampen the overall immune system activation through a decrease in activation, proliferation and cytokine production. Consequently, inhibitory antibodies were developed, such as ipilimumab, blocking CTLA-4 function and thereby facilitating positive co-stimulation with CD28 and allowing the rescue of exhausted CD8 T cells and NK cells, leading to tumor control of colon and melanoma tumors [26]. However, as we will discuss later in this review, anti-CTLA-4 therapy alone is not sufficient to induce a response for all patients, hence the growing focus on new immunotherapeutic targets, particularly PD-1.

### 2.2. PD-1, Programmed Death-1

Programmed death-1 (PD-1, CD279) is a cell surface receptor belonging to the CD28 family of receptors and is commonly seen on T cells and B cells to modulate T cell dysfunction, exhaustion, and tolerance. In addition, PD-1 expression was observed on NK and NKT cell populations. Like CTLA-4, TCR engagement induces the expression of PD-1 on the surface of T cells, leading to a decrease in antitumor cytokines such as IFNγ, survival proteins like Bcl-xL, proliferation, survival and cytotoxicity [31].

PD-1 has two ligands, programmed death-ligand 1 (PD-L1, also called B7-H1; CD274) and programmed death-ligand 2 (PD-L2, also called B7-DC; CD273), whose expression varies according to the cell type and their activation status. PD-L2 has a three-fold higher affinity than PD-L1 and is expressed mainly on DCs, B-cells, macrophages, and monocytes [32], whereas PD-L1 is found on tumor cells, antigen presenting cells, T lymphocytes, endothelial cells and fibroblasts [33].

The intracellular domain of PD-1 contains two tyrosine motifs, immunoreceptor tyrosine-based inhibition motif (ITIM) and immunoreceptor tyrosine-based switch motif (ITSM), which are phosphorylated upon PD-L1/PD-L2 binding. This leads to the recruitment of protein tyrosine phosphatases (PTPs), such as SHP-2 (but also SHP-1), to the cytoplasmic tail of PD-1. SHP2 downregulates TCR co-stimulation via the dephosphorylation of TCR key signaling elements, mainly CD3ζ, ZAP70, PKCθ and PI3K [34,35]. Since PI3K activity is required to activate AKT, PD-1 is also responsible for AKT inhibition. Additionally, it has been suggested that PI3K/AKT inhibition could be mediated by an alternative pathway. It is suspected that PD-1 alters casein kinase 2 (CK2) phosphorylation, resulting in an increase in phosphatase and tensin homolog (PTEN) activity, an important inhibitor of PI3K [36].

Furthermore, it is believed that PD-1 also inhibits RAS-MEK-ERK pathway signaling, inducing T cells growth arrest through SHP-2 dephosphorylation of PLCγ1 [37]. Both PI3K-AKT and RAS-MEK-ERK signaling are required for transcriptional Skp2 expression. Thereby, simultaneous inhibition of PI3K/AKT and Ras signaling by anti PD-1 induces Skp2 suppression associated with p27kip1 degradation. Moreover, anti-PD-1 leads to impairment of CDK2 activation, failing to activate Rb and Smad3 [38] and consequently impairing the cell cycle machinery by the accumulation of p15 (inhibitor of CDK4/6) and repression of cdc25a [38].

Finally, upon PD-1 engagement, PI3K-AKT and ERK inhibition results in PD-1-dependent metabolic alterations [39]. Since the SHP2 pathway has been dissected through numerous in vitro studies, Rota’s team [40] focused on an in vivo approach and concluded that SHP2 is not essential for PD-1 inhibitory signaling. This can be explained by the fact that in the absence of SHP2, redundant mechanisms take over to compensate for the loss [40]. This study clearly shows that further investigation is required to fully understand the intricacy of PD-1 inhibitory functions.

What is certain is that the STAT pathway is regulated strongly and specifically by SHP1 or SHP2. Direct regulation of the STAT pathway by PD-1 has not been carefully studied in T cells during immunotherapy. In fact, STAT5a has been shown to interact specifically with SHP2 [41], while STAT3 is mainly regulated by SHP1 [42]. STAT3 activation results in increased Tregs with increased IL10 production [43]. Additionally, STAT3 phosphorylation increases production of PD-L1 in NKT lymphoma models [44]. From mice models deficient in PD-1, a higher level of phosphorylation of STAT5a was observed in ILCs [45]. Experiments show that PD-1 engagement decreases STAT5a phosphorylation [45]. We can hypothesize that SHP2 could be responsible of this STAT5 regulation, as well as STAT3, through SHP1.

In B cells, PD-1 seems to act differently but with the same aim, which is to dampen the immune system and to negatively regulate B cell activation and proliferation. PD-1 seems to down-regulate IL-6 production [46] and prevent the production of antibodies by B cells [47]. However, circulating antibodies were elevated in PD-1 deficient mice, increasing autoimmune disease susceptibility [48].

Additionally, a high expression of the PD-L1 ligand on B cells’ PD-1+ decreases T cell (CD4 and CD8) proliferation [49]. Recently, B cells have been associated with an increased number of tumoral T cells through B cell production of pro-inflammatory cytokines during PD-1 immunotherapy [50]. Okazaki’s team demonstrated that PD-1 prevented B cell receptor (BCR) transduction by recruiting and phosphorylating tyrosine-protein phosphatase non-receptor type 11 (PTPN11), which in turn dephosphorylates BCR-signaling molecules such as spleen tyrosine kinase (SYK), leading to a decrease in phosphoinositide 3-kinase (PI3K), phosphoinositide-specific phospholipase C γ2 (PLCγ2), and extracellular signal–regulated kinases (ERK) [51].

It has been noted that certain tumors were unresponsive to CD8^+^ T cells due to their low MHC expression. In that case, evidence shows that PD-1/PD-L1 blockade is still effective through NK cell anti-tumor immunity. Indeed PD-1 is believed to be expressed by NK cells and represses their cytotoxicity and anti-tumor response. This event can be partially restored by antibody disrupting PD-1/PD-L1 interaction (Hsu et al., 2018). However, these data were not reproduced in a recent study carried out by Judge and colleagues, who were not able to detect PD-1 expression in a tumor or viral models [52] and concluded that NK cells play a minor contribution during immunotherapy. Despite this, PD-1 expression in NK cells seems to be different than in T cells. PD-1 expression was transient and rapid on splenic NK cells in the early stages of post-cytomegalovirus infection (MCMV) and was dependent on glucocorticoid expression [53]. Better control of conditions and timing need to be developed to study PD-1 blockade on tumoral NK cells. Due to these inconsistencies, NK signaling has not been extensively studied and needs to be addressed due to their nonspecific activation mode and the similarity of the phenotype with treatment with anti-PD-1.

Regardless of those uncertainties, antibodies directed against PD-1/PD-L1 have been developed. While anti-CTLA-4 therapy acts mainly on CD4 during the antigen presentation, anti-PD-1 is more effective at restoring the effector function of exhausted CD8^+^ T cells to clear tumor cells. An increasing body of evidence has shown that anti-PD-1 also acts on B or NK cells to restore their CD8 activity. Successful clinical trials [54,55,56] confirmed PD-1 as the most effective target and today PD-1/PD-L1 checkpoint blockade therapy is part of the standard therapy for multiple malignancies, such as Hodgkin’s lymphoma and melanoma.

### 2.3. LAG-3, Lymphocyte Activation Gene 3

Lymphocyte activation gene 3 (LAG-3, CD223) is a cell surface receptor expressed on activated human T cells, B cells and NK cells [57]. LAG-3 is thought to be associated with CD3 in order to impede T cell proliferation, cytokine production and calcium flux [58,59]. Secondly, this receptor is believed to be implicated in T cell homeostasis [60], cancer, chronic viral infection and parasitic infection. Moreover, further data reveal that PD-1 may act alongside LAG-3 in the regulation of autoimmunity [61].

Around 20% of LAG-3 highly conserved structural motifs are identical to CD4. Based on their structural homology, it has been established that CD4 and LAG-3 share a mutual ligand; major histocompatibility complex class II (MHC class-II) molecules on APCs (HLA-DR, HLA-DQ, HLA-DO, HLA-DP, HLA-DM) [62], with around 100-fold greater affinity for LAG-3 than CD4 [63]. Mutagenesis studies confirmed that LAG-3 interaction with MHC class II involves amino acid residues of the proline-rich D1 loop, a structural feature that is not present in CD4 [64]. Given these observations, LAG-3 was suggested to compete with CD4 for MHC class II binding, thus negatively impacting CD4 function. Human melanoma often expresses MHC class II molecules, and this expression is associated with poor prognosis [65]. Thus, MHC class II ligation of LAG-3 [66] could induce melanoma-infiltrating T cell exhaustion.

The specificity of LAG-3 is its uncommon cytoplasmic tail, not shared with any other immune receptor. It is composed of a serine-phosphorylation site and a unique KIEELE motif, along with a region containing glutamic acid-proline (EP) repeats. Early studies suggested that LAG-3-associated protein (LAP), identified in a yeast two-hybrid screen, may bind to the EP motif [67]. However, mutants lacking the EP motif maintain receptor activity, suggesting that it may not be essential for LAG-3 function [68]. In contrast, the KIEELE motif is described as essential regarding the inhibitory functions of LAG-3 [69]. The singularity of this domain indicates that LAG-3 operates through mechanisms quite distinct from its other co-inhibitory receptor colleagues. A single lysine residue (Lys468) in a mouse sequence was shown to be indispensable and is conserved across all species [70]. This motif in the cytoplasmic tail is indispensable for abrogating effector CD4^+^ T cells. It can prevent the entry of T cells into the proliferation phase, and deletion of this region prevents the negative signal to T cell function, allowing for Il-2 production [69]. Moreover, LAG-3 expression on CD4^+^ T cells modulate cytokines including IL-2, IL-7, IL-12 and IFN-γ [71]. Likewise, LAG-3-deficient (Lag3–/–), CD4^+^ T cells also secrete more cytokines like IL-2 and IFNγ following in vitro stimulation, although with reduced expansion due to increased cell death [60]. Resting CD8^+^ T cells also express low levels of LAG-3, which is strongly upregulated in response to antigenic stimulation [72]. LAG-3 blockade enhances the function of CD8^+^ T cells, which produce more IFN-γ. Moreover, recently FGL1, a protein secreted by liver and cancer cells, was identified as a new ligand of LAG-3, leading to T cells suppression. Anti-LAG-3 or anti-FGL1 antibody treatment allows T cells to be reactivated, leading to a decrease in tumor size [73].

LAG-3 antibodies have no direct effect on human natural killer cytotoxicity compared to T cells [74]. However, increased cytokine secretion (such as IFNγ, TNFα, GMCSF) was observed [75], but LAG-3 has no direct impact on NK cytotoxicity. Yet, LAG-3 plays a more critical role in NKT cells with a down-regulation of the proliferation through arresting S phase in the cell cycle [76]. Moreover, LAG-3 has also been reported to exhaust invariant NKT (iNKT) cells and reduce IFN-γ production in HIV-infected patients [77].

The downstream effector and the intracellular process remain unidentified. Therefore solving the signaling associated with LAG-3 is of great importance. Indeed, various melanoma infiltrating cells exhibit LAG-3 expression [66]. B16-F10 model mice treated with LAG-3-blockading antibody (monotherapy) only exhibited delayed tumor growth, whereas combination therapy targeting both LAG-3 and PD-1 at the time of relapse resulted in significant tumor regression [78]. Recent results in melanoma (phase III RELATIVITY-047 trial) were published at ASCO, revealing that a combination of LAG-3 antibodies with anti-PD-1 increased the progression free survival to 10.1 months compared to nivolumab alone (4.6 months) [79].

### 2.4. TIM-3, T-Cell Immunoglobulin and Mucin-Domain Containing-3

T-cell immunoglobulin and mucin-domain containing-3 (TIM-3) is the receptor for galectin 9, phosphatidyl serine, HMGB1 and ceacam-1, and is known to be expressed by regulatory T (Treg) cells and innate immune cells such as dendritic cells (DCs), natural killer (NK) cells, monocytes, macrophages, and mast cells [57].

On the one hand, it has been reported that under certain circumstances this receptor could play a co-stimulatory enhancing immune function. High expression of TIM-3 on T-cell lines increased activation of transcriptional activity of NFAT/AP-1 and nuclear factor-kappa B (NF-κB) using reporter assays, a higher phospho-S6 and enhanced levels of cytokines [80]. TIM-3 expression is also associated with increased IFN-γ production, but when crosslinked to an antibody it suppresses NK cytotoxicity [81]. On the other hand, TIM-3 was associated with poor prognosis and suppression of anti-tumor function with an inhibitory role in T cells as TIM-3-expressing CD4^+^ and CD8^+^ T cells produce a weak amount of cytokine with less proliferative phenotype in response to antigen stimulation [82,83,84]. In addition, TIM-3 has been found to suppress Th1 and Th17 responses [85,86] and induce peripheral immune tolerance [87,88]. Additionally, TIM-3 expression on T cells is considered a mark of exhaustion during chronic infection and its engagement with galectin 9 can induce apoptosis in T cells. These observations support the inhibitory role of TIM-3 in T cells.

One possible reason for this antagonistic effect could come from ceacam-1 expression. As a matter of fact, it has been suggested that TIM-3 inhibitory function is dependent on the co-expression of ceacam-1 in tumors and autoimmune diseases [89]. Recently, a study from Annika De Sousa Linhares et al. identified Ceacam1 isoform 4L to have an inhibitory effect on transcription factor NF-κB and NFAT on activated T cells [90]. However, this inhibition was independent of TIM-3 expression, and in vitro binding assay between Ceacam-1 and Tim-3 failed. However, they confirmed that the intracellular part of TIM-3 is responsible for the inhibitory effect on NF-κB and NFAT on activated T cells by TCR engagement. Function of TIM-3 and Ceacam-1 remains unclear and needs to be investigated to understand the mechanism of TIM-3 induced inhibition.

TIM-3 cytoplasmic tail is composed of numerous tyrosine residues, unlike the other immune inhibitors that have been shown to be phosphorylated, especially tyrosines 256 and 263 [80], and involved in the binding of Bat3 leading to the preservation and promotion of T cell signaling [91]. Upon ligand engagement, BAT3 is released from TIM-3 tail resulting in T cell inhibition. Furthermore, FYN can bind to the same region on the TIM-3 tail as BAT3 and has been associated with T cell anergy [92]. One possible hypothesis could be that a switch TIM-3/FYN and TIM-3/BAT3 is responsible for the dual inhibitory/activatory function of TIM-3. In any case, the downstream effectors of BAT3 remains unknown. It is interesting to note that the absence of BAT3 was associated with the loss of the ability to produce large amounts of IFN-γ and IL-2 in T cells [93].

TIM-3 has been observed on macrophages where it acts as a negative regulator of the NLRP3 inflammasome by dampening the NF-κB pathway in mouse peritoneal macrophages [94]. As in T cells, tyrosines 256 and 263 are necessary for NLRP3 inhibition by TIM-3. Regarding activated NK cells, TIM-3 is strongly expressed and its ligation with galectin-9 is necessary for IFNγ secretion [95]. On the other hand, its engagement with ligands, including antibodies, subsequently suppresses cell cytotoxicity [81]. This is also the case in advanced melanoma patients, where TIM-3 is found on functionally exhausted NK cells with reduced IFN-γ secretion and cytotoxicity [96].

Finally, TIM-3 is expressed on DCs and has been shown to inhibit their activation and maturation via BTK and c-SRC to prevent NF-κB signaling [97]. Furthermore, TIM-3 blocks the response to TLR3, TLR7, TLR9 and cytosolic sensors to DNA and RNA by interacting with HMGB1 and impairing the recruitment of nucleic acids to endosomes [98]. This TIM-3 mechanism suppresses antitumor immunity mediated by nucleic acids in tumor microenvironments. In addition, TIM-3 was implicated in the dysfunction of plasmacytoid DCs by interfering with TLR signaling via the recruitment of IRF7 and p85 to lysosomes during chronic HIV infection [99]. In contrast, an activating effect is seen on mast cells. TIM-3 is constitutively expressed on these cells and enhances FcεRI signaling, leading to degranulation and cytokine release after antigen binding, confirming the co-stimulatory function of TIM-3 [100]. Subsequently TIM-3 was considered an immunotherapy candidate and the administration of TIM-3 and PD-1 monoclonal antibodies displayed a synergic controlled melanoma tumor growth [101]. Since then, combination with immunotherapies has been tested in different cancers, showing improvements in immunotherapy outcomes [102].

### 2.5. TIGIT, T Cell Immunoglobulin and ITIM Domain

T cell immunoglobulin and ITIM domain (TIGIT) is an inhibitory receptor, and a newly identified member of the CD28 family. TIGIT was found to be strictly expressed on T cell subsets (including Tregs and memory T cells), along with NK cells, and applies direct immunosuppressive effects to these cells [103,104].

TIGIT binds two ligands, CD112 (nectin-2, also known as PRR2 or PVRL2) and, with much higher affinity, CD155 (PVR or Necl-5), both identified on APCs, T cells and a variety of non-hematopoietic cell types including melanoma cells [57]. It is interesting to note that CD155 and CD112 are over-expressed in several cancers, including melanoma [105]. Similarly to the CTLA-4/CD28 dynamic, TIGIT outcompete DNAM-1 (CD226) and CD96 for the binding of CD155, impeding DNAM-1 positive co-stimulation and delivering a direct inhibitory signal [103,104,106] as shown in TIGIT-deficient mice with delayed tumor growth [107]. TIGIT can also indirectly suppress T cell activation by a TIGIT–CD155 interaction on DCs. This binding leads to the inhibition of IL-12 production and increases the secretion of IL-10, consequently promoting tolerogenic DCs that downregulate T cell responses [104].

Regarding the signaling of TIGIT, the different studies available focused mainly on NK cells. Indeed, TIGIT seems to exert a negative effect through its cytoplasmic ITIM and ITT domain with cytotoxicity, granule polarization and cytokine release inhibition [108]. Upon receptor engagement, the ITT-like motif becomes phosphorylated, binds β-arrestin 2 and recruits SHIP1 to limit NF-κB signaling, leading to the suppression of IFN-γ production [109,110]. The binding of cytosolic adapter growth factor receptor-bound protein 2 (Grb2) also promotes SHIP1 recruitment that it responsible for PI3K and MAPK inhibition [108]. On T cells, the engagement of TIGIT is supposed to target TCR signaling, such as PLCγ, and down-regulate IFNγ secretion as well as IL-17 [106,111]. In contrast, TIGIT is also presumed to be responsible for the up-regulation of anti-apoptotic molecules such as Bcl-xL as well as induction of the receptors for IL-2, IL-7 and IL-15 implicated in T cell survival. A possible explanation would be that TIGIT maintains the T cell repertoire and keeps them from anergy while repressing their activation.

The general review of available literature highlighted the remaining gaps in TIGIT’s story. The question of whether nectin-3 is another ligand for TIGIT is still unresolved [103]. Moreover, TIGIT-CD112 interaction still needs to be addressed. Even though several unanswered questions persist, targeting TIGIT in a clinical setting could have great therapeutic potential. TIGIT expression was found to be higher in the cells within tumor microenvironment than in those in the periphery, which could theoretically mean that using anti-TIGIT monoclonal antibody would provide a more specific targeted immunotherapy with less autoimmune-related toxicities. Along these lines, experiments using TIGIT−/− mice suggested that targeting TIGIT could potentially trigger fewer immune-related adverse events (irAEs) than anti-PD-1 or anti-CTLA-4 [112]. However, TIGIT blockade with either anti-PD-1/PD-L1 or TIM-3 showed a synergistic effect with a slowdown of tumor growth [107,113] but no remission was observed.

### 2.6. VISTA, V-Domain Ig Suppressor of T Cell Activation

V-domain Ig suppressor of T cell activation (VISTA), also known as PD-1 homolog (PD-1H), is an immune checkpoint regulator expressed on hematopoietic cells (neutrophils, macrophages, T-cells) [114] with the ability to suppress the activity of T cells [115]. Studies establish that VISTA not only acts as a repressive ligand of T cell activation and proliferation and cytokine production, but also as a stimulatory immune ligand for APCs [116,117]. Interestingly, blocking VISTA on APC cells leads to T cell activation [115]. Therefore, VISTA appears to be a unique dual molecule functioning as both a receptor and a ligand. In addition, VISTA was found to be highly homologous to PD-1 [115] except for its conserved cytoplasmic domain composed of two potential protein kinase C binding sites and a proline rich motif, which may be its potential docking sites. Lately, VSIG-3 was proposed to be the novel ligand for VISTA [118], acting together in an inhibition function toward T cells. The VISTA/VSIG-3 combination remains to be observed in vivo. The multiple studies available highlight VISTA heterogeneity with a variation of expression according to the tissue and tumor type. Despite the prediction difficulties associated with its variability, blocking VISTA has proven to be effective. Indeed, VISTA blockade enhanced the infiltration, proliferation, and effector function of tumor-infiltrating T cells within the TME, even when VISTA expression is low within tumor cells [119]. Accordingly, VISTA is considered a potential candidate to improve actual immunotherapies with more tumor specificity and less irAEs, much like TIGIT.

### 2.7. B7H3, B7 Homolog 3

B7 homolog 3 (B7-H3), also named CD276, is a protein that belongs to the B7-CD28 pathway family, constitutively expressed on murine APCs and on activated human T cells [120], NK cells, DCs, macrophages and monocytes. Initially, B7-H3 was described as a positive co-stimulator of T cell responses and IFN-γ production [121]. However, studies have established its inhibitory function toward T cell activation, proliferation and cytokine production [122,123,124]. More distinctively, B7-H3 was found to be overexpressed in multiple malignancies, including melanoma [125], and associated with bad prognoses. Indeed, B7-H3 was implicated in cancer aggressiveness since it was shown to modulate migration, invasion and adhesion to the fibronectin of various cancer cells [126], including melanoma cells, with the regulation of metastasis-associated proteins MMP-2, TIMP-1, TIMP-2, STAT3 and IL-8 [123]. To date, no receptor has been clearly identified, and B7-H3 may have more than one binding partner with distinct functions that could explain its complex immunomodulation. What is clear is the potential value of targeting the B7-H3 checkpoint seen as this protein evidently plays a role in tumor immunity.

B7-H3 inhibition control tumor growth and anti-tumor immunity is dependent on CD8^+^ T cells and natural killer (NK) cells. Moreover, B7-H3 directly inhibits the activation of NK cells. Cotreatment with anti-B7-H3 and anti-PD-1 increases tumor regression in hepatocellular carcinoma compared to PD-1 monoclonal antibody treatment alone [127].

## 3. Therapeutic Strategies Targeting Immune Checkpoints

The discovery of regulatory molecules in the immune system over the past 15 years has completely changed therapeutic strategies in the treatment of cancers. This new approach has led to the clinical development of therapeutic monoclonal antibodies blocking CTLA-4 and the PD-1/PD-L1 axis. Several studies have demonstrated promising results as patients with different tumor types presented relevant objective response rates and extended the overall progression free survival results. Indeed, ipilimumab (Yervoy^®^), a CTLA-4 inhibitor, has shown efficiency in the reconstitution of the anti-tumor immune response and was given FDA approval in 2011 for the treatment of melanoma [128]. The anti-PD-1 agents pembrolizumab (Keytruda^®^) and nivolumab (Opdivo^®^) displayed even better clinical results and toxicity profiles, leading to their FDA approval in 2014. Unfortunately, the benefits of monotherapy were limited to only a fraction of all patients. In fact, 30% of patients treated with anti-PD-1 and only 10–15% of patients treated with anti-CTLA-4 have a complete response. The others only have partial tumor regression or no response to monotherapy at all. More recently, combination approaches were proposed to increase patient response and survival rates. Currently, the rate of objective response to immunotherapy for melanoma has reached 52% for pembrolizumab [129] and 58% for the combination of nivolumab and ipilimumab compared to 45% in the nivolumab group and 19% in the ipilimumab group alone [130]. In addition, the five year overall survival rate was increased with combined therapy, 41% for pembrolizumab, 52% for the nivolumab-plus-ipilimumab group and 44% for the nivolumab group compared to the 26% rate for the ipilimumab alone group. Despite these encouraging results, a subset of patients remains resistant, and on top of that, the patients on a combination of CTLA-4 and PD-1 therapy are faced with severe toxicities. The need to explore new therapeutic angles is becoming more pressing. With that in mind, new immune checkpoint blockade combinations have been the focus of recent research and are presented in Table 1.

In vivo data demonstrate that the single knockout of PD-1 or LAG-3 in mice shows a subtle and limited result whereas the blockade of dual LAG-3/PD-1 reveals a clear synergy between these two molecules with a significant melanoma and colon tumor regression [131]. The same results were found regarding TIM-3, TIGIT, VISTA and B7-H3 inhibition pathways [101,113,119,132]. As described above, these molecules were reported to be highly expressed in immune cells in the TME, especially on TILs and Tregs. Targeting them would allow a more effective reactivation of CD8 T lymphocyte-specific function. In line with these findings, several drugs directed against those newly identified inhibitory receptors were developed and their combination with anti-PD-1 is currently being tested with the aim of increasing the efficacy by extending progression free and overall survival while reducing toxicity. These compounds and the clinical trials associated with them are summarized in Table 1.

Preliminary data concerning combination treatment using anti-LAG-3 (BMS-986016, relatlimab) and anti-PD-1 (nivolumab) predict encouraging outcomes with a 16% overall response rate and a 45% disease control rate, especially considering that this study was performed on patients with melanoma who relapsed or were refractory to anti-PD-1/PD-L1 therapy [133]. Furthermore, the LAG-3/PD-1 combination had a similar safety profile to nivolumab monotherapy.

Presently, therapeutic antibodies are a major tool used to block immune checkpoint and reactivate exhausted or dampened immune cells by tumor cells. Despite their high specificity, their use implies high immunogenicity, which may cause side effects, low tumor penetration, and high treatment costs. On that account, peptides and small chemical compounds were considered to overcome those issues. Lately, a novel cyclic peptide, C25, targeting LAG-3, has been developed and is currently under clinical trial. Hopefully, this molecule will overcome previous limitations or support actual antibody-based therapies.

From these encouraging results and trials, growing evidence suggests that males and females respond differently to immune checkpoint inhibitors. Interestingly, animal studies have shown that estrogen upregulates PD-1 and PD-L1 expression [134,135]. Along with this observation, a more recent study found that PD-L1 blockade was more effective in female mice than in male mice [136]. In 2018, a meta-analysis [137] of twenty clinical trials in subjects with advanced or metastatic cancers treated with immune checkpoint inhibitors showed significant sex differences. Males had a poorer overall survival compared to women treated with immune checkpoint inhibitors. Furthermore, melanoma patients demonstrate greater sex differences in their response to immune checkpoint inhibitors compared to patients with non-small cell lung cancer. In particular, overall survival of patients treated with anti CTLA4 were more influenced by sex difference compared to those treated by anti PD-1 [138]. According to these results, sex hormone therapy could be of interest in combination with ICIs but further research is required to carefully investigate the relationship between hormones and response to immunotherapy. Moreover, individuals’ sex hormone levels evolve with age and modify one’s immune system activity, further contributing to the complexity of the assessment of complement therapy. Aging itself is an important factor in one’s therapeutic response. Defects in mismatch repair increases with age and could lead to differences in responses to immunotherapy. Several studies have pointed out that tumor deficiencies in effective mismatch repair presented a better response to immunotherapy. These studies have highlighted that those tumors with deficient repair have increased the amount of mutant peptide presented in their T cells [139] and high genomic instability, and such mutations will lead to effective responses to anti PD-1 [140], with an increase in overall survival [141]. In melanoma tumors, Hugo et al. found that a high tumor burden was significantly associated with overall survival in responders but was not associated with their response to immunotherapy. Highly mutated neoantigens were thought to be responsible for the efficacy of immune checkpoints until recently, when Changzheng Lu et al. [142] demonstrated an accumulation of cytosolic DNA fragment and production of IFNβ in a cGAS-STING dependent manner in a MLH1 deficient tumor cell model (defective mismatch repair), sensitizing patients to immune checkpoint therapy. Guan Junhong et al. shows that MLH1 negatively regulates Exo1 exonuclease activity. Thus, loss of MLH1 results in uncontrolled DNA excision by Exo1, which causes increased ssDNA formation, DNA breaks, and aberrant DNA repair, leading to chromosomal instability and cytosolic DNA accumulation. The latter activates the cGAS-STING pathway to facilitate immunotherapy and IFNβ production [143].

This implication of DNA in activating immune cells gives us a strong rationale for using chemotherapy or targeted therapy prior to or during immune checkpoint therapy. Chemotherapy or targeted therapy will lead to cell death associated with antigen release and fragmented DNA in the microenvironment, rendering the tumor more susceptible to the immune system. This approach could help in poorly mutated tumors that do not respond to ICI. In NSCLC, use of cisplatin prior to anti-PD1 treatment allows for the presentation of non-mutated neoantigens in apoptotic cells, increasing the efficacy of anti-PD1 treatment, which was corelated with patient survival [144]. In melanoma, targeted therapy associated with immune checkpoint inhibitors are currently undergoing clinical trials. A study of a French cohort [145] highlighted the necessity of investigating this approach in the case of BRAF WT tumors. In this study, patients treated with MEKi and anti-PD1 had an 83% disease control rate and a progression free survival of 7.1 months while BRAF-mutated patients undergoing the same treatment only had 2.5 month progression free survival. Several clinical trials using combined chemotherapy or targeted therapy with immunotherapy are ongoing and results are expected soon.

The failure of individual immune checkpoint responses relies on a number of different mechanisms. Either cells had innate resistance or were able to acquire resistance through different mechanisms. These mechanisms may be varied, from specific mutations to the reprogramming of tumor cells leading to immune escape. In PD-1 resistant patients, several mutations have been shown to induce resistance, including B2M and JAK1/JAK2 [146]; however, the reprogramming of tumor cells is the most frequent. Hugo et al. identified a specific signature in melanoma that is associated with resistance to therapy and called it an IPRES signature [147]. This signature is mainly composed of genes involved in EMT (such as AXL TWIST2), immunosuppressive genes (CCL2, CCL8) and interestingly, a number of genes that are regulated by MITF, the master regulator of melanocyte differentiation that is also implicated in melanoma migration, survival and stemness. This aspect is developed in [148].

More recently, B-catenin was involved in resistance to combination therapy (anti CTLA4 + anti-PD1) [149]. The authors showed a lack of infiltration of T cells in tumors, presenting the activation of the WNT/β-catenin pathway. Using a mouse model presenting activated β-catenin, they identified CCL4 as being downregulated, decreasing T cell attraction to the tumors.

## 4. Resistance to PD-1 Blockade, ITGBL1 a New Immune Checkpoint?

To date, despite these hopeful advances in immunotherapy efficiency, a large group of patients remain refractory to treatment and still do not experience durable responses due to their acquisition of resistance. The mechanisms underlying this lack of responsiveness are considered highly multifactorial, [150] with different determinants of resistance such as dysfunction of effector cells, that can be overcome by ICI, and generation of immunosuppressive TME. 

Among TME changes, hypoxia regulates tumor immunity by acting on T cells, NK cells and remodeling the microenvironment to an immunosuppressive state [151,152]. PD-L1 as well as CTLA-4 [153,154] have been shown to be upregulated under hypoxia, decreasing ICI efficacy.

In several aggressive cancers, such as melanoma, numerous studies have demonstrated a switch of tumor cells from an “epithelial” to a “mesenchymal” state, enhancing tumor migration and ICI resistance [155,156]. One protein that is critical to the phenotypic plasticity and resistance mechanisms in melanoma is the transcription factor MITF, the master regulator of melanocyte homeostasis [157,158,159]. Furthermore, MITF impacts immune function [148]. Interestingly, we recently showed that MITF controls the expression of ITGBL1. Integrin beta-like 1 (ITGBL1) was first identified from an osteoblast cDNA library as a β integrin related protein. Its N-terminal EGF-like stalk fragment is highly homologous to β integrin but does not display a transmembrane domain or an RGD (Arg–Gly–Asp)-binding domain, suggesting that ITGBL1 performs distinct functions compared to other β integrins. In breast cancer, non-small cell lung cancer, ovarian cancer and colorectal cancer, ITGBL1 induces cell migration, invasion and adhesion. More recently, this protein has also been linked to melanoma. As a matter of fact, Hugo’s team performed an RNAseq analysis on tumors from patients resistant to anti-PD-1 and extracted a list of genes that are predictive of resistance [147]. Among upregulated genes, inflammatory, wound healing and angiogenesis genes, which are considered T cell-suppressive, were found, as well as genes implicated in the epithelial–mesenchymal transition, along with ITGBL1. From a functional point of view, we have recently demonstrated, for the first time, that ITGBL1 regulates immune function. ITGBL1 has been characterized as a secreted protein allowing melanoma cells to escape immune surveillance by inhibiting NK cell cytotoxicity [160]. Further studies are currently underway to try and understand the precise mechanisms involved in this inhibition, which may be targeted for specific and more efficient targeting of the immune system.

## 5. Conclusions

In conclusion, the identification of new immune checkpoints, like ITGBL1, is necessary to improve patient treatment. Moreover, the presence of immune checkpoint receptors at the surface of different immune cell populations may act differently and might inhibit responses to immunotherapy or increase efficiency. It is of utmost importance that the immune crosstalk and contribution of each separate immune cell population under specific immune checkpoint inhibitors is fully understood. Further studies are needed to understand their precise function as well as the mechanisms of these new immune checkpoints and their receptors by dissecting intracellular signaling and identifying new effectors. Finally, clinical results from ongoing combination treatment assays of different ICIs combinations will give new hope to patients who are unresponsive to current therapies, like those observed with a combination of a MEK inhibitor and a BRAF inhibitor, to overcome resistance of melanoma to targeted therapy.

## Figures and Tables

**Table 1:** Different compounds and their combinations in clinical trials. +/− indicates a study performed with or without a specified compound, mAb: monoclonal antibody, IgG: immunoglobulin G.

Immune Checkpoint	Compound Name	Combination	Phase	Stage	ClinicalTrials.gov ID
LAG-3	**IMP-321** LAG-3Ig fusion protein and APC activator	Pembrolizumab	I	Unresectable or Metastatic Melanoma	NCT02676869
		Tumor Antigenic Peptides and Montanide	I/II	Stage II-IV Melanoma	NCT01308294
		Immunological peptides and immunological adjuvants + HLA-A2 peptides + Montanide ISA51	I/II	Disease-Free Melanoma	NCT00365937
		Cyclophosphamide, fludarabine, Melan-A VLP vaccine	I	Metastatic Melanoma	NCT00324623
		+/− Avelumab	I	Solid Tumors	NCT03252938
	**Relatlimab** **(BMS-986213)** Fully human IgG4 mAb	Nivolumab	I	Advanced Solid Tumors	NCT03335540
			II	MSI-H Solid Tumors	NCT03607890
				Stage IIIB/IV Melanomas	NCT02519322
				Locally Advanced, Unresectable, or Metastatic Melanoma	NCT03724968
			II/III	Advanced Melanoma	NCT03470922
		+/− Nivolumab	I	Advanced Solid Tumors	NCT02966548
			I/II	Solid Tumors Including Melanoma	NCT01968109
			II	Metastatic Melanoma	NCT03743766
		Nivolumab + rHuPH20	I	Cancer including Melanoma	NCT04112498
		Nivolumab + BMS-986205 + Ipilimumab	I/II	Solid Cancers	NCT03459222
		Ipilimumab	I	Advanced Melanoma	NCT03978611
	**Ieramilimab (LAG525)** Fully human IgG4 mAb	PDR001	I/II	Advanced Solid Tumor Including Melanoma	NCT02460224
		PDR001, capmatinib, canakinumab, ribociclib	II	Previously Treated Unresectable or Metastatic Melanoma	NCT03484923
	**MK-4280** Fully human IgG4 mAb	Pembrolizumab + Oxaliplatin+ Irinotecan + Leucovorin (Calcium Folinate) + Fluorouracil [5-FU] + MK-4280A	I	Advanced Solid Tumors	NCT02720068
	**REGN3767** Fully human mAb	+/− cemiplimab	I	Advanced Cancers	NCT03005782
	**TSR-033** Fully human IgG4 mAb	Anti-PD-1	I	Advanced Solid Tumors	NCT03250832
	**BI 754111** Humanized IgG4 mAb	BI 754091(anti-pd1)	Early Phase I	Neoplasms	NCT03433898
			II	Advanced Solid Tumors	NCT03697304
		+/− BI 754091(anti-pd1)	I	Advanced Cancers	NCT03156114
		BI 754091(anti-pd1) + BI907828	I	Advanced Solid Tumors	NCT03964233
	**Sym022** Fully human Fc-inert mAb	-	I	Advanced Solid Tumor	NCT03489369
		Sym021 (PD1)	I	Advanced Solid Tumor	NCT03311412
	**FS118** Bispecific antibody binding both LAG-3 and PD-L1	-	I	Advanced and Metastatic Cancer	NCT03440437
	**MGD013** Bispecific DART protein binding both LAG-3 and PD-1	+/− margetuximab (anti-HER2)	I	Advanced Solid Tumors	NCT03219268
	**INCAGN02385** Fc engineered IgG1k antibody	-	I	Advanced Malignancies	NCT03538028
TIM-3	**TSR-022** Humanized mAb	TSR-042, TSR-033	I	Advanced Solid Tumors	NCT02817633
		Niraparib, TSR-042, Bevacizumab, Platinum-Based chemotherapy	I	Advanced Solid Tumors	NCT03307785
	**MBG453** Humanized IgG4 monoclonal antibody	PDR001	I/II	Advanced Malignancies.	NCT02608268
	**Sym023** Fully human mAb	-	I	Advanced Solid Tumor	NCT03489343
		Sym021, Sym022	I	Advanced Solid Tumor or Lymphomas	NCT03311412
	**INCAGN2390** mAb	-	I	Advanced Malignancies	NCT03652077
	**LY3321367** mAb	LY3300054	I	Advanced Solid Tumor	NCT03099109
		LY3300054, Ramucirumab, Abemaciclib, Merestinib	I	Advanced Solid Tumor	NCT02791334
	**BMS-986258** Fully human mAb	Nivolumab, rHuPH20	I/II	Advanced Solid Tumor	NCT03446040
	**SHR-1702**	Camrelizumab	I	Advanced Solid Tumor	NCT03871855
	**RO7121661** Anti-PD-1/TIM3 bispecific Ab	-	I	Advanced Solid Tumor	NCT03708328
	**BGB-A425** Humanized, IgG1-variant monoclonal antibody	Tislelizumab	I/II	Locally Advanced or Metastatic Solid Tumors	NCT03744468
	**LY3415244** Anti PD-L1/TIM-3 Bispecific Antibody	-	I	Advanced Solid Tumor	NCT03752177
TIGIT	**MK-7684** IgG1 mAb	Pembrolizumab	I	Advanced Solid Tumor	NCT02964013
	**Etigilimab/OMP-313 M32** mAb	Nivolumab	I	Advanced Solid Tumor	NCT03119428
	**Tiragolumab/MTIG7192A/RG-6058** Fully human IgG1 mAb	Atezolizumab	I	Advanced Solid Tumor	NCT02794571
	**BMS-986207** IgG1 mAb (FcyR-null)	Nivolumab	I/II	Advanced Solid Tumor	NCT02913313
	**AB-154** Fully humanized IgG1 mAb	AB122	I	Advanced Malignancies	NCT03628677
	**ASP-8374** IgG1 mAb (FcyR-null)	Pembrolizumab	I	Advanced Solid Tumors	NCT03260322
		-	I	Advanced Solid Tumor	NCT03945253
VISTA	**JNJ-61610588** Complete human mAb	-	I	Advanced Solid Tumor	NCT02671955
	**CA-170d** Small orally available molecule that directly targets PD-L1/PD-L2, and VISTA	-	I	Advanced Solid Tumors	NCT02812875
B7-H3	**Enoblituzumab/MGA271** Humanized IgG1κ mAb	-	I	Advanced Solid Tumors	NCT01391143
		Ipilimumab	I	Advanced Solid Tumors	NCT02381314
		Pembrolizumab	I	Advanced Solid Tumors	NCT02475213
		-	I	Children with B7-H3-Expressing Solid Tumors	NCT02982941
	**MGD009** Humanized, bispecific DART molecule that recognizes both B7-H3 and CD3	MGA012	I	Advanced Solid Tumors	NCT03406949
		-	I	B7-H3-Expressing Tumors	NCT02628535
